# Skin Strain Analysis of the Scapular Region and Wearables Design

**DOI:** 10.3390/s21175761

**Published:** 2021-08-26

**Authors:** Arianna Carnevale, Emiliano Schena, Domenico Formica, Carlo Massaroni, Umile Giuseppe Longo, Vincenzo Denaro

**Affiliations:** 1 Department of Orthopaedic and Trauma Surgery, Campus Bio-Medico University, Via Álvaro del Portillo, 00128 Rome, Italy; g.longo@unicampus.it (U.G.L.); denaro@unicampus.it (V.D.); 2 Unit of Measurement and Biomedical Instrumentation, Campus Bio-Medico University, Via Álvaro del Portillo, 00128 Rome, Italy; e.schena@unicampus.it (E.S.); c.massaroni@unicampus.it (C.M.); 3 Unit of Neurophysiology and Neuroengineering of Human Technology Interaction (NeXT), Campus Bio-Medico University, Via Álvaro del Portillo, 00128 Rome, Italy; d.formica@unicampus.it

**Keywords:** wearable systems, textile sensors, strain sensors, scapula movements monitoring, skin strain analysis, rehabilitation, biomechanics

## Abstract

Monitoring scapular movements is of relevance in the contexts of rehabilitation and clinical research. Among many technologies, wearable systems instrumented by strain sensors are emerging in these applications. An open challenge for the design of these systems is the optimal positioning of the sensing elements, since their response is related to the strain of the underlying substrates. This study aimed to provide a method to analyze the human skin strain of the scapular region. Experiments were conducted on five healthy volunteers to assess the skin strain during upper limb movements in the frontal, sagittal, and scapular planes at different degrees of elevation. A 6 × 5 grid of passive markers was placed posteriorly to cover the entire anatomic region of interest. Results showed that the maximum strain values, in percentage, were 28.26%, and 52.95%, 60.12% and 60.87%, 40.89%, and 48.20%, for elevation up to 90° and maximum elevation in the frontal, sagittal, and scapular planes, respectively. In all cases, the maximum extension is referred to the pair of markers placed horizontally near the axillary fold. Accordingly, this study suggests interesting insights for designing and positioning textile-based strain sensors in wearable systems for scapular movements monitoring.

## 1. Introduction

Patients suffering from shoulder musculoskeletal disorders (MSDs) may experience pain and reduced functional capacity [1,2]. The scapula, the bone linking the humerus with the clavicle, ensures proper alignment and the normal mobility of the glenohumeral and acromioclavicular joints [3]. A correct and coordinated scapular movement represents the key component in regular shoulder functionality. Alterations in scapular position and orientation, a condition known as scapular dyskinesis, characterize most shoulder MSDs, such as subacromial impingement syndrome, rotator cuff tears, frozen shoulder, or multidirectional instability [1,4]. The scapulothoracic joint is a functional sliding joint between the medial border of the scapula and the posterior thoracic ribcage, allowing the relative motion of the scapula on the thoracic surface below. The joint variables in sliding joints are the extensions between two sequential body segments [5,6].

Obtaining objective data of the scapular movements considering both different degrees of elevations and planes (e.g., frontal, sagittal, and scapular) could provide meaningful achievements in the context of rehabilitation and clinical research [7,8,9]. Recently, increasing attention has been directed toward understanding the complex scapula kinematics and providing monitoring systems that can quantify scapular movements [8,10]. To date, several techniques for the analysis of scapular kinematics have been successfully proposed [11,12,13,14]. Traditionally, the gold standard for kinematic analysis are techniques using fluoroscopy, bone pins, or optical motion capture systems [15,16,17]. Although these techniques are accurate and highly reliable for kinematic analysis, they are expensive, time-consuming, and too laborious to be applied in standard clinical practice and unstructured environments [18]. Moreover, in the last few decades, wearable systems for human joints motion monitoring have been employed for sports, healthcare, and security applications. Such systems can integrate several typologies of sensors, among which magnetic-inertial measurement units (M-IMUs), inertial measurement units (IMUs), textile-based and fiber optic-based sensors [9,19,20,21,22,23]. However, the design of comfortable wearable systems for applications outside of a structured laboratory environment still presents some open challenges. In this regard, wearable systems based on textile strain sensors represent an emerging solution [6]. These sensors can be an excellent solution to monitor scapular sliding [9,24]. They enjoy several advantages, including flexibility, adaptability to different anthropometries, easy integration into stretchable skin-tight garments. The correct positioning of the sensing elements directly on the skin or on a garment is crucial. Some studies placed the textile sensors on garments for scapular movements detection, relying on empirical selections [8,25]. Although this approach may be valid for simple joints, more accurate methods should be investigated to determine the optimal placement and orientation of the textile sensors. This precaution is especially important in the case of complex joints as the scapulothoracic one. Indeed, the sensors’ outputs will depend on the attachment points and how the underlying skin deforms as a result of the motion. No previous works have preceded the development of a wearable system integrating textile-based strain sensors for scapula motions monitoring with a skin strain analysis. Previous works investigated the human skin strain around the knee joint [26,27], upper body [28], and ankle-foot complex [29], aiming at developing wearable systems (e.g., tight garments, orthotics). Only [30] investigated skin strain of the shoulder joint to guide the development of a mechanical counter-pressure space suit, using three-dimensional digital image correlation.

The overall goal of this study is to provide a method to analyze the human skin strain of the scapular region. By evaluating the skin strain, wearable textile-based strain sensors could be developed as a second skin-like for monitoring scapular movements. The specific aims of this study were: (i) to develop a method for analyzing human-skin strain of the scapular region using an optical motion capture system recording 3D displacements of a grid of retro-reflective markers, and (ii) to estimate how skin deformation varies in the scapular region when performing upper limb movements at different degrees of elevation in the frontal, sagittal, and scapular planes.

## 2. Scapular Movements and Effects of Underlying Soft Tissue on Strain Distribution

Although this section does not deal exhaustively with the anatomy and biomechanics of the scapula, a greater focus has been placed on the soft tissues underlying the posterior scapular region. Indeed, muscles, adipose, and connective tissues, in turn, influence superficial skin deformation during upper limb movements. The development of a wearable system based on textile strain sensors aiming at monitoring scapular movements cannot disregard the knowledge of the biomechanics of the scapulothoracic joint, which is among the most complex in the human body.

**Figure 1:**
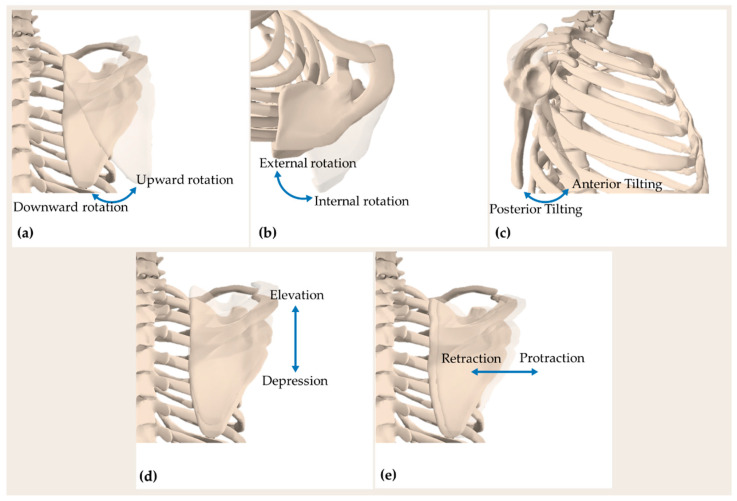
Movements of the scapula: (**a**) posterior view showing scapula upward-downward rotation; (**b**) superior view showing scapula internal-external rotation; (**c**) lateral view showing scapula anterior-posterior tilting; (**d**) posterior view showing scapula elevation-depression; and (**e**) posterior view showing scapula retraction-protraction. All representations refer to a right scapula.

Biomechanically, scapular movements are a combination of translations and rotations that occur simultaneously and not in isolation during upper arm elevation, justifying the complexity of monitoring scapular movements with wearable systems. The three rotational degrees of freedom of the scapulae include (i) upward-downward rotation around an axis perpendicular to the scapular plane, (ii) internal-external rotation around a vertical axis through the scapular plane, and (iii) anterior-posterior tilting around an axis along the scapular spine [31]. Translations include superior-inferior (elevation-depression) and mediolateral (retraction-protraction) motions of the scapulae over the posterior chest wall. Translational movements are permitted by the connection of the scapula to the axial skeleton through the clavicle [31]. Figure 1 illustrates the main scapular movements.

**Figure 2:**
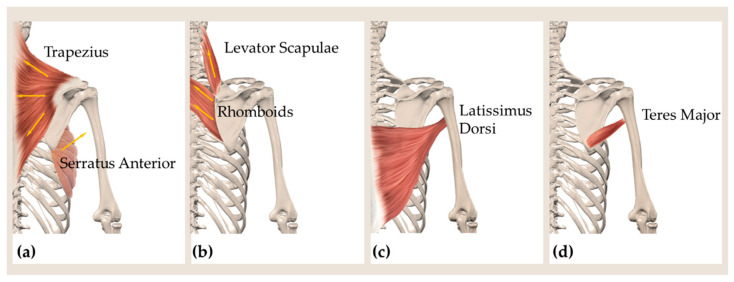
Drawing showing the main lines of action of (**a**) the muscles trapezius (upper, middle, lower fibers), serratus anterior; (**b**) rhomboids and levator scapulae. Posterior view showing the location of the muscles latissumus dorsi (**c**) and teres major (**d**), constituting the posterior border of the axillary region.

The scapula serves as the location of various muscles’ attachment [3]. Such muscles, having different sizes, functions, and depths, experience several stretching directions during upper limb movements in the different planes of the 3D space and at different degrees of elevation. Moreover, the scapula posteriorly is covered by overlying soft tissue, which in turn influences the superficial deformation of the scapular region. For all of these reasons, the skin deformations in the scapular region have dissimilarity in stretching position and magnitude during upper limbs motions. The main scapulothoracic muscles are the trapezius muscle, the serratus anterior muscle, the rhomboids, and the levator scapulae [3]. During active flexion and abduction of the shoulder, the trapezius act as scapular retractor, and the serratus anterior enables the upward rotation and protraction of the scapulothoracic joint [3,32,33]. The rhomboids and levator scapulae mainly contribute to the scapula’s retraction, elevation, and internal rotation [3,10]. In Figure 2a,b, a schematic representation of the main lines of action of the aforementioned muscles is presented.

The axillary fold is located below the glenohumeral joint connecting the humerus to the glenoid fossa of the scapula. In addition to being the site of a certain amount of fatty tissue and connective tissue, the axillary region posteriorly borders with the latissum dorsi muscle (Figure 2c) and teres major muscle (Figure 2d). The latissimus dorsi muscle is part of the muscles of the scapular movements enabling inferior angle pulling in multiple directions. Indeed, its multidirectional muscle fibers allow shoulder adduction, extension, and internal rotation. Besides allowing the movements (internal rotation and extension) of the humerus at the glenohumeral joint, the teres major muscle contributes to the scapular upward rotation and elevation [3].

## 3. Materials and Methods

### 3.1. Participants

In this study, five male volunteers (mean ± standard deviation: age—25.4 ± 3.8 years old; body mass—74.8 ± 9.6 kg; height—1.77 ± 0.11 m; body mass index—23.7 ± 1.9 kg∙m^−2^) with no history of shoulder pathologies were recruited. All participants performed the experimental tasks with their dominant (right) limb. Before experimental sessions, all subjects read and signed an informed consent, approved by the Ethical Committee of University Campus Bio-Medico of Rome (protocol code: 09/19 OSS ComEt UCBM).

Table 1 shows the age and main anthropometric characteristics of the subjects involved in the study.

### 3.2. Experimental Set-Up

**Figure 3:**
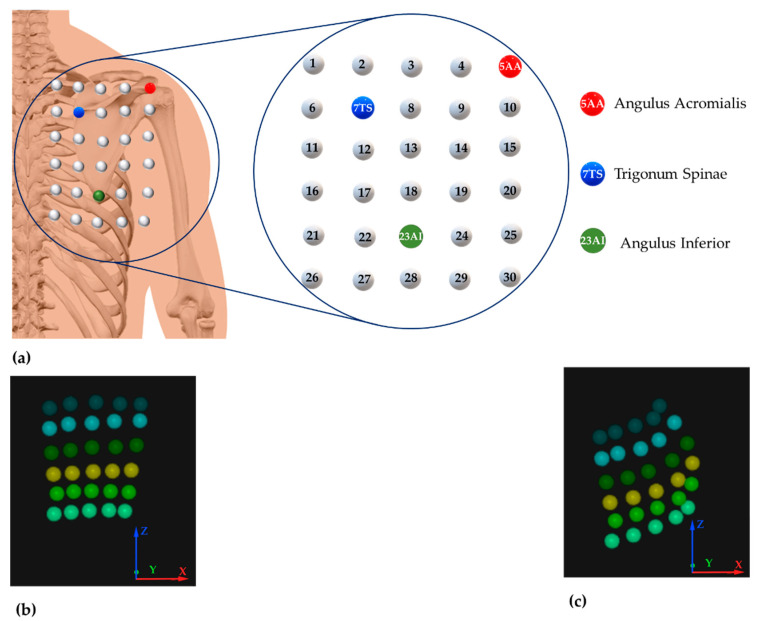
(**a**) Schematic representation of the 6 × 5 grid of markers positioned on the skin surface of the scapular region. (**b**) Reconstruction of the grids of markers corresponding to the starting and (**c**) elevated position.

A Qualysis™ Motion Capture system (Qualysis AB, Gothenburg, Sweden) equipped with 10 Miqus M3 cameras (sampling frequency, 100 Hz) and 2 Miqus Video (sampling frequency, 25 Hz) was used to track a 6 × 5 grid of spherical retro-reflective markers (diameter, 8 mm). All markers were positioned on the right scapular region by the same investigator to avoid bias. Firstly, three markers were positioned on three skeletal landmarks of the scapula, i.e., angulus acromialis, trigonum spinae, and angulus inferior, identified by surface palpation. Then, the remaining 27 markers were positioned to form the 6 × 5 grid covering the entire scapular region of each subject (Figure 3a). Figure 3b,c show an actual reconstruction of the grid of markers during a task performed by a volunteer representing the starting position and elevated position, respectively.

### 3.3. Experimental Protocol

Volunteers were verbally instructed by the same investigator, who also provided a practical demonstration of each task to be performed.

**Figure 4:**
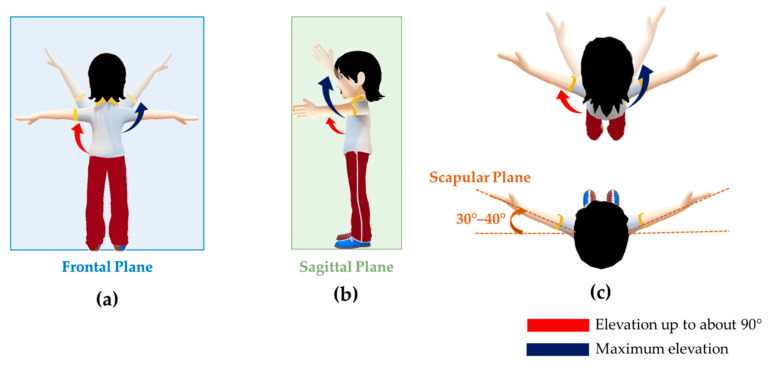
Experimental protocol: (**a**) elevation in the frontal plane; (**b**) elevation in the sagittal plane; (**c**) elevation in the scapular plane. The red and blue arrows represent the elevation up to 90° and the maximum elevation, respectively.

During experimental sessions, the starting position was with the arms along the body and palms towards the thighs. Figure 4 illustrates the movements investigated during experiments.

Volunteers were invited to perform the following six tasks:

1. Task 1: 10 consecutive arm abductions in the frontal plane from starting position to approximately 90°.
2. Task 2: 10 consecutive arm abductions in the frontal plane from starting position to maximum elevation.
3. Task 3: 10 consecutive arm flexions in the sagittal plane from starting position to approximately 90°.
4. Task 4: 10 consecutive arm flexions in the sagittal plane from starting position to maximum elevation.
5. Task 5: 10 consecutive arm elevations in the scapular plane from starting position to approximately 90°.
6. Task 6: 10 consecutive arm elevations in the scapular plane from starting position to maximum elevation.

All tasks were executed with the elbow fully extended and the thumb pointing upward. During each task, the same investigator guided the participants to perform the movements.

### 3.4. Data Analysis

#### 3.4.1. Motion Capture Data

The collected data were first pre-processed off-line using the Qualisys Track Manager (QTM) software (version 2021.1, Build 6300) for markers’ labeling and trajectories gap filling by applying proprietary algorithms included in QTM software. All gap-filled trajectories were visually inspected. For further analysis, a process of manual identification of events corresponding to the starting and elevation positions reached by volunteers at each repetition was performed in QTM. Then, data of all subjects and executed tasks were exported to MATLAB (version 2020b). Markers’ trajectories data were filtered using a low pass 4th order Butterworth filter with a cutoff frequency of 6 Hz. As there is no consensus on the directionality of deformation experienced in the scapular region during upper limb elevations, distances between pairs of markers were not calculated separately in the vertical and horizontal directions. Instead, distances between pairs of markers were computed by considering all possible combinations (i.e., 435) considering 30 elements (i.e., the number of markers) taken 2 at a time.

For each pair of markers, the distance D(i,j) between the i−th marker m(i) and the j−th marker m(j) was obtained as:

(1) $$D\left(i,j\right)=\sqrt{\sum_{i\neq j}{\left(m\left(i\right)-m\left(j\right)\right)}^{2}}\;\mathrm{with}\;i,\;j=1,\;\ldots ,\;30$$

#### 3.4.2. Skin Deformation Analysis and Statistics

**Figure 5:**
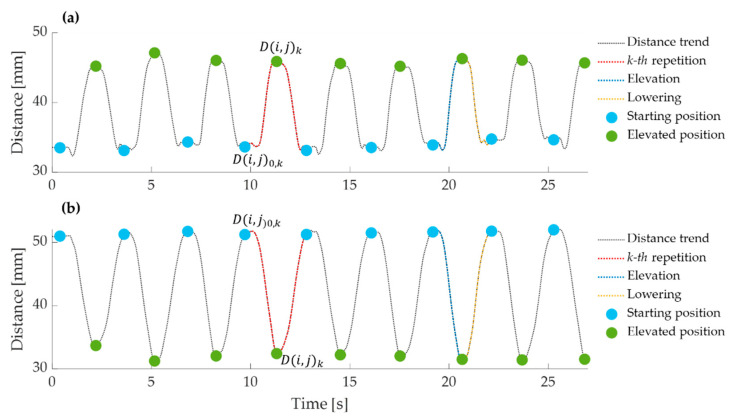
Two examples of distance trend (in black) between pairs of markers collected on a volunteer during a trial. The ten repetitions are visible. The distance during a k−th repetition is highlighted in red, during an elevation phase in blue, and during a lowering phase in yellow. The events corresponding to the starting and elevated positions are highlighted by light blue and green circles, respectively. (**a**) Example of distance increasing during the elevation phase (in blue) and decreasing during lowering (in yellow), which means a skin extension, and (**b**) example of distance decreasing during the elevation phase (in blue) and increasing during the lowering (in yellow) which means a skin compression.

For each pair of markers (i,j) with i≠j, the skin relative strain variation $\varepsilon_{k}\left(i,j\right)$ at each k−th repetition was calculated using the following equation:

(2) $$\varepsilon_{k}\left(i,j\right)=\frac{D{\left(i,j\right)}_{k}-D{\left(i,j\right)}_{0,k}}{D{\left(i,j\right)}_{0,k}}=\frac{\Delta D{\left(i,j\right)}_{k}}{D{\left(i,j\right)}_{0,k}}\;\mathrm{with}\;k=1,\ldots ,\;10$$

where $D{\left(i,j\right)}_{k}$ and $D{\left(i,j\right)}_{0,k}$ are the distances between the i−th and j−th markers corresponding to the k−th repetition at the elevated position and starting positions, respectively, and $\Delta D{\left(i,j\right)}_{k}$ is the difference between the two mentioned distances. For greater clarity, Figure 5 shows the events corresponding to the starting position (light blue circle) and to the elevated position (green circle) for each repetition (in red).

The mean percentage strain, $\bar{\varepsilon }\%$, was calculated as follows:

(3) $$\bar{\varepsilon }\%=\frac{\sum_{k=1}^{10}\varepsilon_{k}}{10}\times 100$$

A positive value of the mean strain $\bar{\varepsilon }\%$ corresponds to the skin extension, while a negative value corresponds to the skin compression.

After calculating $\bar{\varepsilon }\%$, variations in skin strain were averaged among the five participants for each pair of markers. The descriptive analysis was performed by evaluating mean, median, standard deviation, minimum, and maximum strain. The Shapiro-Wilk test was used to assess the normality assumption of the data. If the Shapiro-Wilk test results were significant (*p* < 0.05), the nonparametric Wilcoxon rank-sum test was applied as a statistical method for strain comparison at 90° and maximum elevation in all planes. For all hypothesis tests, the *p*-value for significance was 0.05 for the rejection of the null hypothesis. Statistical analysis was performed in SPSS v28.0 (IBM, SPSS, Inc., Chicago, IL, USA).

## 4. Results

**Figure 6:**
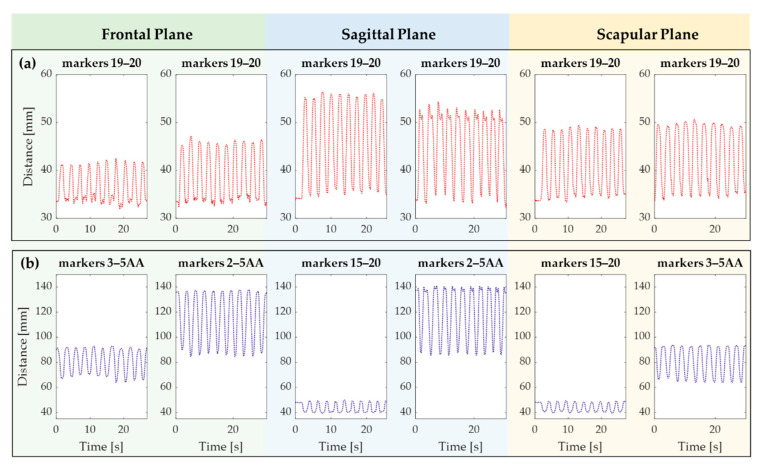
The distance trends recorded on a volunteer during the six tasks. (**a**) Example of pairs of markers showing skin extension and (**b**) skin compression during the elevation phase.

A total of 435 skin relative strain variations in the scapular region from 5 participants were analyzed during arm elevation in the frontal, sagittal, and scapular planes at 90° and maximum degree of elevation. During the elevation phase in all planes and at different degrees, some pairs of markers moved away, and others moved closer, suggesting extension and compression of the underlying scapular region, respectively. Figure 6 reports the distance trends of some pairs of markers during all tasks performed by a volunteer.

The Shapiro-Wilk test showed that strain distributions corresponding to different degrees of elevation were not normally distributed (Table 2). Moreover, the differences between strain at 90° and maximum elevation were significant, as shown by the results of the Wilcoxon rank-sum test (*p* < 0.05), see Table 2.

**Figure 7:**
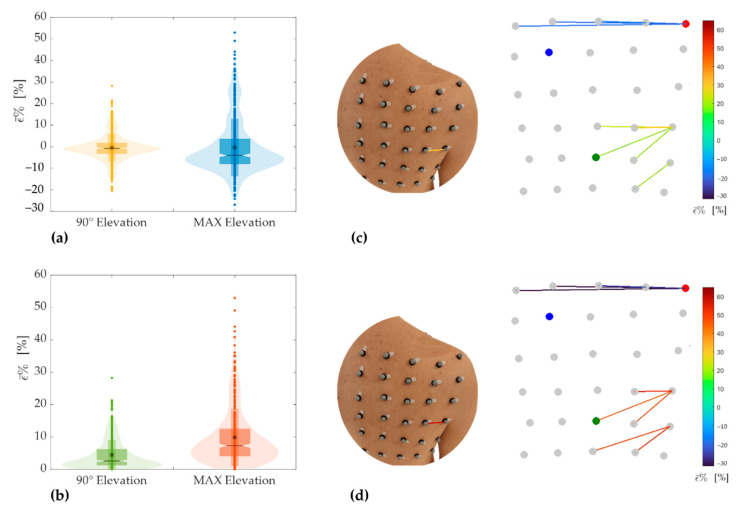
Violin and box plots showing strain distribution (**a**) and distribution of the absolute strain values (**b**) corresponding to the tasks performed in the frontal plane. Maximum extension and compression extreme values corresponding to the elevation in the frontal plane up to 90° (**c**) and maximum elevation (**d**).

Figure 7a reports the combination of box and violin plots to provide in a single representation the main features of strain distributions during the tasks performed in the frontal plane. The box plot allowed highlighting the mean value (represented by the asterisk), the median value (represented by the black horizontal line), and the interquartile range, IQR (represented by the upper and lower limits of the box plots) of $\bar{\varepsilon }\%$. The violin plot allowed showing the $\bar{\varepsilon }\%$ distribution of all the 435 pairs of markers (shaded area) and all of the 435 $\bar{\varepsilon }\%$ values (dots).

From the analysis of Figure 7a is clear the greater dispersion of the $\bar{\varepsilon }\%$ during the task at maximum elevation (in blue) than task up to about 90° (in yellow). For maximum elevation in the frontal plane, results of $\bar{\varepsilon }\%$ showed a mean ± standard deviation equals to −0.36 ± 13.27, a median of −4.05, and an IQR of −8.10–3.58. For 90° of elevation in the frontal plane, the mean ± standard deviation was −0.46 ± 6.43, the median was −0.80, and the IQR was −3.39–1.86. The bigger extension of the IQR calculated during maximum extension confirms the higher dispersion in this task.

A similar analysis has been performed considering the $\bar{\varepsilon }\%$ absolute values reported in Figure 7b. Such analysis allows comparing the $\bar{\varepsilon }\%$ experienced during the two degrees of elevation by focusing on the skin strain’s amplitude without discriminating between compression and extension. From the analysis of Figure 7b, it is clear that during the task at maximum elevation (in orange), the absolute value of $\bar{\varepsilon }\%$ is bigger than the one up to about 90° (in green). For maximum elevation in the frontal plane, the mean ± standard deviation was 9.90 ± 8.84, the median was 7.30, and the IQR was 4.02–12.56. For 90°, the mean ± standard deviation was 4.46 ± 4.64, the median was 2.60, and the IQR was 1.21−6.23. These results highlight that skin strains in the scapular region are greatest during maximal abduction and are also confirmed by the maximum $\bar{\varepsilon }\%$ value (i.e., 52.95% for maximum elevation vs. 28.26% for elevation up to 90°). The region that underwent maximum extension corresponds to the pair of markers 19–20 for both degrees of elevation in the frontal plane (Figure 7c,d).

The region that underwent maximum compression during upper arm abduction corresponds to the first line of the grid for both degrees of elevation (Figure 7c,d).

Table 3 reports the extreme values of $\bar{\varepsilon }$% for both extension and compression during tasks performed in the frontal plane. Data confirm that skin strains are bigger during maximal abduction.

**Figure 8:**
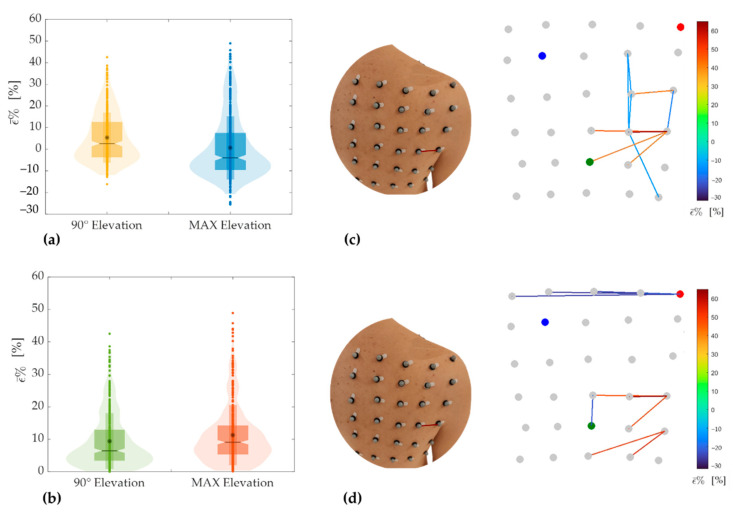
Violin and box plots showing strain distribution (**a**) and distribution of the absolute strain values (**b**) corresponding to the tasks performed in the sagittal plane. Maximum extension and compression values corresponding to the elevation in the sagittal plane up to 90° (**c**) and maximum elevation (**d**).

From the analysis of Figure 8a is clear the greater dispersion of the $\bar{\varepsilon }\%$ during the task at maximum elevation (in blue) than task up to about 90° (in yellow) performed in the sagittal plane. For maximum elevation in the sagittal plane, results of $\bar{\varepsilon }\%$ showed a mean ± standard deviation equals to −6.87 ± 14.62, a median of −3.96, and an IQR of −9.68–7.50. For 90°, the mean ± standard deviation was −5.37 ± 11.59, the median was −2.55, and the IQR was −3.77–12.56.

Also in this case, from the analysis of Figure 8b is clear that during the task at maximum elevation (in orange) the absolute value of $\bar{\varepsilon }\%$ is bigger than the one up to about 90° (in green). For maximum elevation in the sagittal plane, the mean ± standard deviation was 11.30 ± 9.28, the median was 9.10, and the IQR was 5.32–14.23. For 90°, the mean ± standard deviation was 9.40 ± 8.64, the median was 6.47, and the IQR was 3.39–12.86.

The maximum positive values were found to be 60.87% and 60.12% for maximum and 90° of elevation, respectively (Table 4). The region that underwent maximum extension corresponds to the pair of markers 19–20 for both degrees of elevation in the sagittal plane (Figure 8c,d). Unlike movements performed in the frontal plane, during upper arm elevations in the sagittal plane, the pairs of markers corresponding to the maximum compressive strain values were not distributed along the same direction (Figure 8c,d). Table 4 reports the extreme values of $\bar{\varepsilon }$% for both extension and during tasks performed in the sagittal plane.

**Figure 9:**
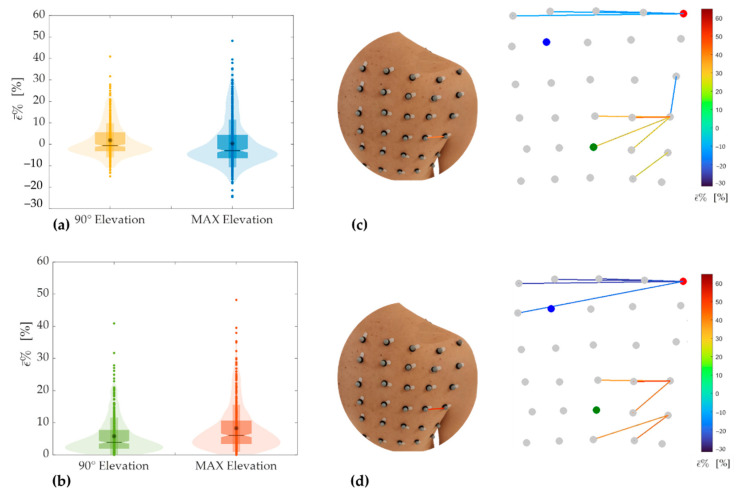
Violin and box plots showing strain distribution (**a**) and distribution of the absolute strain values (**b**) corresponding to the tasks performed in the scapular plane. Maximum extension and compression values corresponding to the elevation in the scapular plane up to 90° (**c**) and maximum elevation (**d**).

Figure 9a shows strain distributions of the scapular region corresponding to the elevations performed in the scapular plane. For maximum elevation in the scapular plane, results of $\bar{\varepsilon }\%$ showed a mean ± standard deviation equals to 0.32 ± 11.08, a median of −2.96, and an IQR of −6.56–4.39. For 90° of elevation in the scapular plane, the mean ± standard deviation was 1.86 ± 7.94, the median was −0.60, and the IQR was −3.22–5.57.

As in the two previous planes, from the analysis of Figure 9b is clear that during the task at maximum elevation (in orange) in the scapular plane, the absolute value of $\bar{\varepsilon }\%$ is bigger than the one up to about 90° (in green). For maximum elevation in the scapular plane, the mean ± standard deviation was 8.28 ± 7.36, the median was 6.05, and the IQR was 3.34–10.70. For 90°, the mean ± standard deviation was 5.79 ± 5.74, the median was 3.87, and the IQR was 1.91–7.76.

The maximum positive values were 48.20% and 40.89% for maximum and 90° of elevation, respectively (Table 5). The region that underwent maximum extension corresponds to the pair of markers 19–20 for both degrees of elevation in the scapular plane (Figure 9c,d). As in the case of movements performed in the sagittal plane, also during the elevation of the upper limb in the scapular plane, the distribution of the pairs of markers corresponding to the maximum compressive strain values is not concentrated along the same row of the grid of markers. Even in this case, the region that underwent greater extension was the one surrounding the axillary fold, although along slightly different directions than in the other planes (Figure 9c,d). Table 5 reports the extreme values of $\bar{\varepsilon }$% for both extension and compression during tasks performed in the scapular plane.

## 5. Discussion

Monitoring scapular movements may be useful in rehabilitation and clinical research. This study proposes a methodological approach to quantify scapular skin strain using a 6 × 5 grid of retro-reflective markers. We implemented this method for upper limb flexion in the sagittal plane, elevation in the scapular plane (scaption), and abduction in the frontal plane. This analysis may be fundamental for the development of some solutions able to monitor the scapular movements. Indeed, an open challenge in the development of wearable systems based on strain sensors is the proper placement of the sensing elements. To date, several textile-based strain sensors have been designed and employed to measure human joints movements [7,8,22,34,35,36,37]. Among textile-based strain sensors, resistive ones are popular for instrumenting wearables [6,19]. These sensors are mainly composed of an elastic textile substrate and conductive materials, which undergo microstructural changes in response to an applied deformation resulting in electrical resistance variation in the sensing elements [6,7]. The textile component enables the integration into garments as adherent as possible or into polymeric substrates that could potentially be directly applied to the skin. The textile component allows the sensitive element to stretch and relax during movements, thanks to its elastic characteristics. One of the main requirements for developing wearable systems integrating textile-based strain sensors is that they should adhere perfectly to the surface of the body region of interest. Moreover, improper orientations of the sensors could negatively influence the sensitivity for joints movements detection. As regard scapular movements, the unreliable reading of textile-based strain sensors is further influenced by the simultaneity of translations and rotations that the scapula undergoes during upper limb movements. For this reason, identifying the areas in the scapular region that experience the greatest deformation could provide useful information about the design, integration, and placement of textile-based wearable strain sensors. In a previous study [30], skin strain field analysis in the region surrounding the shoulder joint was performed using three-dimensional image correlation technique. Shoulder abduction and flexion were investigated in a single volunteer, showing that the area that experienced more significant strains corresponds to that surrounding the axillary fold posteriorly, in accordance with our findings. Unlike our study, in [13], a grid of markers was placed on the scapular region to obtain a surface mapping from which to infer the scapular kinematics.

In the present study, the motion tracking data were used to provide the distribution of length changes in the posterior scapular region, calculated in terms of distance between all possible combinations of markers pairs. Strain distribution (Figure 7, Figure 8 and Figure 9) shows interesting characteristics for all movements performed in all planes and degrees of elevation. Namely, the region with the highest extension was the area surrounding the axillary fold. Although this region corresponds to an area with a greater amount of underlying soft tissue, it also has a high number of muscles, which contract during arms elevation, inducing a corresponding surface deformation. Results showed a significant difference between elevation up to 90° and maximum elevation for all the performed tasks. Concerning the positive strains (i.e., extension), the highest percentage positive strain was found to be: 28.26% and 52.95% for elevation in the frontal plane up to 90° and maximum elevation, respectively; 60.12% and 60.87% for elevation in the sagittal plane up to 90° and maximum elevation, respectively; and 40.89% and 48.20% for elevation in the scapular plane up to 90° and maximum elevation, respectively. In all these cases, the maximum extension is referred to the pair of markers 19–20 placed horizontally near the axillary fold (see Figure 3). Conversely, the same generality of results cannot be applied to regions that underwent maximum compression. Although the regions subjected to the greatest compression mostly correspond to the first rows of the marker grid (Figure 7c,d, Figure 8c and Figure 9c,d), in some cases, the pairs of markers that experienced the greatest compression are arranged in different regions (Figure 8c,d and Figure 9c). The reason for these results is probably related to the anthropometric heterogeneity of the subjects involved in the experimental trials. This aspect is not of particular relevance in the design of wearable systems based on resistive textile sensors since they work better in extension than in shortening [34]. Therefore, the regions subjected to higher stretch values should be considered for the placement of textile-based strain sensors.

The absence of deep analysis on the skin strain in the scapular region is highlighted by the different positioning and number of resistive textile-based strain sensors used in wearable systems designed for monitoring scapular movements [7,8,22]. Although these studies showed promising results about monitoring scapular movements in healthy subjects and patients with musculoskeletal or neurological disorders, they all empirically placed the sensors on the scapular region.

## 6. Conclusions

In conclusion, this study proposed a new method for skin strain analysis of the scapular region. The method was used to estimate the skin scapular surface strain on five volunteers during upper limb movements of clinical relevance. This is the first study investigating skin deformation of the scapular region induced by arms elevation in different planes and at different degrees of elevation. The results suggested interesting insights for the integration and positioning of resistive textile-based strain sensors within wearable systems for monitoring scapular movements.

## Figures and Tables

**Table 1:** Age, Body Mass, Height, and BMI (Body Mass Index) of the five volunteers.

Volunteer	Age [years]	Body Mass [kg]	Height [m]	BMI [kg∙m^−2^]
V1	24	81	1.73	27.1
V2	24	83	1.89	23.2
V3	25	67	1.72	22.6
V4	32	62	1.63	23.3
V5	22	81	1.90	22.4

**Table 2:** Descriptive statistics (mean, standard deviation, median, min, max of strain), results of the test for normality (Shapiro-Wilk), and test for difference between strain distribution at 90° and maximum elevation (Wilcoxon rank-sum test) performed in the frontal, sagittal, and scapular plane.

$\mathrm{Variable}\;\bar{\varepsilon }\%$	Mean [–]	SD [–]	Median [–]	Min [–]	Max [–]	SW *p*-Value	W *p*-Value
Fro90	−0.46	6.43	−0.80	−20.54	28.26	<0.001 ^a^	0.002 ^b^
FroMax	−0.36	13.27	−4.05	−30.72	52.95	<0.001 ^a^	
Sag90	5.37	11.59	2.55	−16.16	60.12	<0.001 ^a^	<0.001 ^b^
SagMax	0.69	14.62	−3.96	−25.52	60.87	<0.001 ^a^	
Scap90	1.86	7.94	−0.60	−14.91	40.89	<0.001 ^a^	<0.001 ^b^
ScapMax	0.32	11.08	−2.96	−24.54	48.19	<0.001 ^a^	

Fro90: 90° elevation in the Frontal plane; FroMax: maximum elevation in the Frontal plane; Sag90: 90° elevation in the Sagittal plane; SagMax: maximum elevation in the Sagittal plane; Scap90: 90° elevation in the Scapular plane; ScapMax: maximum elevation in the Scapular plane; SD: Standard Deviation; Min: minimum; Max: Maximum; SW: Shapiro-Wilk test; W: Wilcoxon rank-sum test. ^a^ Indicates no normal distribution; ^b^ Indicates statistical significance.

**Table 3:** Extreme percentage values of the mean strain $\bar{\varepsilon }\%$ in both extension and compression during tasks performed in the frontal plane.

	$\mathrm{Frontal}\;\mathrm{Plane}\text{—}\bar{\varepsilon }\%\mathrm{Extreme}\;\mathrm{Values}$			
	Elevation 90°		Max Elevation	
	Pair of Markers	$\bar{\varepsilon }\%\;$	Pair of Markers	$\bar{\varepsilon }\%$
Extension	19–20	28.26	19–20	52.95
	25–29	21.32	25–29	49.07
	18–20	21.16	25–28	44.08
	20–24	20.97	20–24	42.65
	20–23AI	20.00	20–23AI	40.90
Compression	3–5AA	−20.54	2–5AA	−30.72
	4–5AA	−19.70	3–5AA	−30.51
	2–5AA	−18.95	1–5AA	−30.44
	1–5AA	−18.37	4–5AA	−26.92
	3–4	−16.14	3–4	−24.17

AI: Angulus Inferior; AA: Angulus Acromialis.

**Table 4:** Extreme percentage values of the mean strain $\bar{\varepsilon }\%$ in both extension and compression during tasks performed in the sagittal plane.

	$\mathrm{Sagittal}\;\mathrm{Plane}\text{—}\bar{\varepsilon }\%\mathrm{Extreme}\;\mathrm{Values}$			
	Elevation 90°		Max Elevation	
	Pair of Markers	$\bar{\varepsilon }\%$	Pair of Markers	$\bar{\varepsilon }\%$
Extension	19–20	60.12	19–20	60.87
	18–20	42.51	25–29	48.91
	14–15	38.63	25–28	45.77
	20–23AI	37.48	18–20	44.06
	20–24	36.91	20–24	43.74
Compression	15–20	−16.16	2–5AA	−25.52
	9–14	−12.83	1–5AA	−24.95
	9–19	−12.31	3–5AA	−24.44
	14–19	−12.15	4–5AA	−22.06
	19–30	−12.05	18–23AI	−21.63

AI: Angulus Inferior; AA: Angulus Acromialis.

**Table 5:** Extreme percentage values of the mean strain $\bar{\varepsilon }\%$ in both extension and compression during tasks performed in the scapular plane.

	$\mathrm{Scapular}\;\mathrm{Plane}\text{—}\bar{\varepsilon }\%\mathrm{Extreme}\;\mathrm{Values}$			
	Elevation 90°		Max Elevation	
	Pair of Markers	$\bar{\varepsilon }\%$	Pair of Markers	$\bar{\varepsilon }\%$
Extension	19–20	40.89	19–20	48.20
	18–20	31.69	25–29	39.52
	20–23AI	27.84	20–24	37.93
	20–24	27.03	25–28	35.34
	25–29	26.15	18–20	35.13
Compression	15–20	−14.91	3–5AA	−24.54
	4–5AA	−13.35	2–5AA	−24.45
	3–5AA	−13.08	1–5AA	−24.04
	2–5AA	−12.00	4–5AA	−21.43
	1–5AA	−11.57	5AA-6	−18.16

AI: Angulus Inferior; AA: Angulus Acromialis.

## Data Availability

The data presented in this study are available on request from the corresponding author.
